# The key role of repeated DNAs in sex chromosome evolution in two fish species with ZW sex chromosome system

**DOI:** 10.1186/1755-8166-5-28

**Published:** 2012-06-01

**Authors:** Marcelo de Bello Cioffi, Eduard Kejnovský, Vinicius Marquioni, Juliana Poltronieri, Wagner Franco Molina, Débora Diniz, Luiz Antonio Carlos Bertollo

**Affiliations:** 1 Departamento de Genética e Evolução, Universidade Federal de São Carlos, São Carlos, SP, Brazil; 2 Department of Plant Developmental Genetics, Institute of Biophysics ASCR, Brno, Czech Republic; 3 Laboratory of Genome Dynamics, CEITEC - Central European Institute of Technology, Masaryk University, Brno, Czech Republic; 4 Departamento de Biologia Celular e Genética, Centro de Biociências, Universidade Federal do Rio Grande do Norte, Natal, RN, Brazil; 5 Departamento de Ciências Biológicas, Universidade Estadual do Sudoeste da Bahia, Jequié, BA, Brazil

**Keywords:** Microsatellites, Sex chromosome evolution, Heterochromatin, Fish, ZW systems

## Abstract

Despite substantial progress, there are still several gaps in our knowledge about the process of sex chromosome differentiation. The degeneration of sex-specific chromosome in some species is well documented, but it is not clear if all species follow the same evolutionary pathway. The accumulation of repetitive DNA sequences, however, is a common feature. To better understand this involvement, fish species emerge as excellent models because they exhibit a wide variety of sex chromosome and sex determining systems. Besides, they have much younger sex chromosomes compared to higher vertebrates, making it possible to follow early steps of differentiation. Here, we analyzed the arrangement of 9 repetitive DNA sequences in the W chromosomes of 2 fish species, namely *Leporinus reinhardti* and *Triportheus auritus*, which present well-differentiated ZZ/ZW sex system, but differ in respect to the size of the sex-specific chromosome. Both W chromosomes are almost fully heterochromatic, with accumulation of repeated DNAs in their heterochromatic regions. We found that microsatellites have strongly accumulated on the large W chromosome of *L. reinhardti* but not on the reduced-size W chromosome of *T. auritus* and are therefore important players of the W chromosome expansion. The present data highlight that the evolution of the sex chromosomes can diverge even in the same type of sex system, with and without the degeneration of the specific-sex chromosome, being more dynamic than traditionally appreciated.

## Background

Sex chromosomes and their differentiation are among the most interesting topics in evolutionary genetics. However, although evolutionary processes shaping sex chromosomes are still not completely understood the cessation or the partial restriction of recombination within the sex chromosome pair is always observed. Data from phylogenetically distinct organisms show that this phenomenon is frequently associated with the accumulation of repetitive DNAs in the sex chromosomes [1-8], indicating that this feature is an inherent property of sex chromosome differentiation. Repetitive DNA sequences constitute the major fraction of eukaryote genomes and include the tandem repeats (satellites, minisatellites, and microsatellites) and dispersed elements (transposons and retrotransposons) [9,10]. Repetitive DNA plays an important role on the structural and functional organization of genomes [11,12].

Sex chromosomes of birds (ZZ/ZW) and mammals (XX/XY) are highly differentiated, resulting from a long evolutionary process. It is estimated, for example, that the mammalian Y chromosome has been differentiated more than 150 million years [13]. In turn, sex chromosomes of amphibian and fish have a more recent origin, with less than 10 million years in some species [14]. This makes fish, the oldest vertebrate group, a good model for analyzing the evolution of sex chromosomes in vertebrates, since this issue can be followed from the absence of sex chromosomes to the different steps of their differentiation, improving the understanding of the association of repetitive DNA with this event.

Among the most differentiated sex chromosomes in fishes are the ZW ones. *Leporinus* (Anostomidae, Characiformes) represents a frequently investigated genus that shows a conserved chromosome number (2n = 54) with a conspicuous ZW sex chromosome system shared by several species. The typical W chromosome is always the largest one in the karyotype, almost fully heterochromatic and much bigger than the Z chromosome, representing a model of well-differentiated ZW sex systems without degeneration of the sex-specific chromosome [15-20]. On the other hand, *Triportheus* (Characidae, Characiformes) is also a well investigated genus, in which all species present 2n = 52 chromosomes and a ZW sex chromosome system The size of W chromosome in *Triportheus* is reduced compared to the Z chromosome, representing a distinct model of a well-differentiated ZW system with degeneration of the sex-specific chromosome [21-24].

In this study, we compared the degree of repetitive DNAs accumulation on the differentiated W chromosomes in two ZW-fish models - *Leporinus* and *Triportheus*. We found that microsatellites have strongly accumulated on the large W chromosome of *L. reinhardti* but not on reduced-size W chromosome of *T. auritus* and are therefore important players of the W chromosome expansion.

## Results

**Figure 1:**
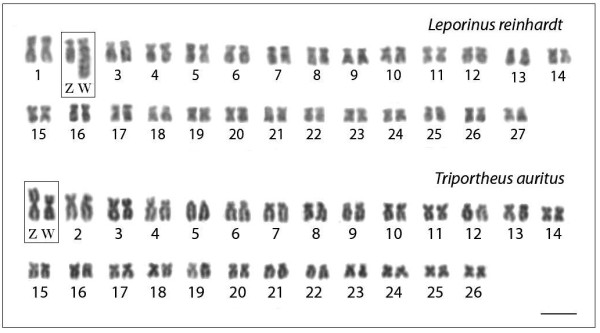
**Giemsa-stained female karyotypes of* Leporinus reinhardti *(2n = 54) and* Triportheus auritus *(2n = 52), both with a ZZ/ZW sex chromosome system.** The chromosomes of both species were arranged in descending order of size and the sex chromosomes were highlighted in boxes for the sake of clarity. Bar = 5 μm.

**Figure 2:**
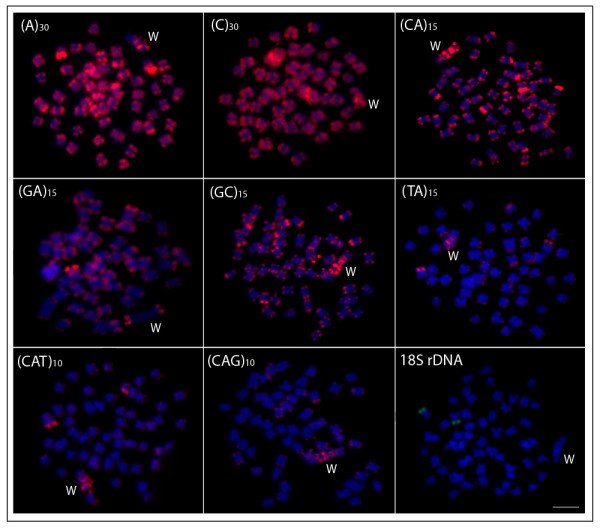
**Mitotic metaphase chromosomes of* Leporinus reinhardti *female, with a ZZ/ZW sex chromosome system hybridized with different repeated DNAs, including mono-, di- and trinucleotide microsatellites and an 18S rDNA gene as probes.** Letters mark the W chromosomes. Bar = 5 μm.

**Figure 3:**
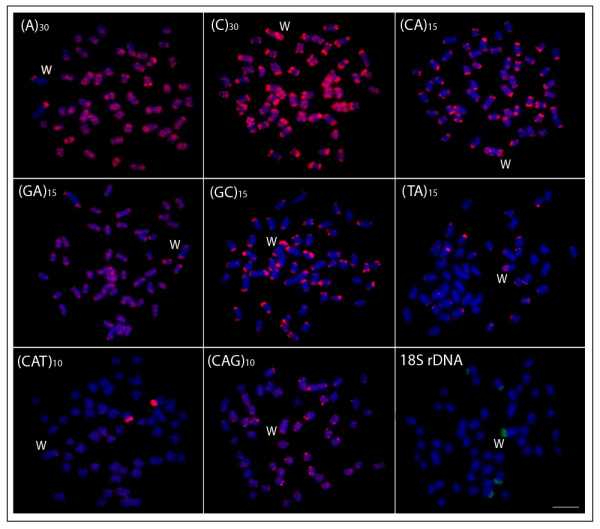
**Mitotic metaphase chromosomes of* Triportheus auritus *female, with a ZZ/ZW sex chromosome system hybridized with different repeated DNAs, including mono-, di- and trinucleotide microsatellites, and an 18S rRNA gene as probes.** Letters mark the W chromosomes. Bar = 5 μm.

**Figure 4:**
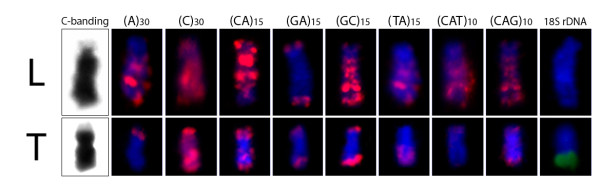
**W chromosomes of* Leporinus reinhardti *(L) and* Triporteus auritus *(T) after C-banding and FISH with various repetitive DNA sequences.** Note the huge accumulation of several classes of microsatellites in *L. reinhardti* and the lesser amount of this accumulation in *T. auritus.*

*Leporinus reinhardti* has a karyotype structure composed of 2n = 54 m-sm chromosomes, with the exception of the W chromosome, while *T. auritus* has 2n = 52 chromosomes comprising m, sm, st and a pairs (Figure 1). Both species presented a distinct heteromorphic ZZ/ZW sex system. In *L. reinhardti*, the sex specific W chromosome is the largest one in the complement and the unique classified as st, allowing it to be easily distinguished from the other chromosomes of the karyotype. On the other hand, in *T. auritus* the Z chromosome is the largest m in the karyotype, while the W is also m, but smaller than the Z (Figure 1). The W chromosomes of both species showed a more highly distinct pattern than the Z chromosome and all autosomal pairs concerning the microsatellite repeats distribution (Figures 2, 3). They are also widely heterochromatic, showing extensive regions of C-positive heterochromatin (Figure 4).

The W chromosome of *L. reinhardti* differ significantly by the strong accumulation of most microsatellite repeats, mainly on its long arm, contrasting with the pattern generally found for the autosomes (Figures 2, 4). Microsatellites (A)_30_, (C)_30_, (CA)_15_, (GC)_15_, (TA)_15_, (CAT)_10_, (CAG)_10_ showed high accumulation on the W chromosome, with the majority of signals being accumulated on its long arm. While the microsatellites (A)_30_, (CA)_15_ and (GC)_15_ were also accumulated on the heterochromatic subtelomeric regions of several autosomes, the (TA)_15_, (CAT)_10_, (CAG)_10_ signals outside the W chromosome were absent or only minor. On the other hand, microsatellite (C)_30_ was uniformly spread along all other chromosomes. Microsatellite (GA)_15_ represented an exception, exhibiting no accumulation on the W chromosome and being found only in telomeres. The 18S rDNA site was restricted to the terminal region of only one autosomal pair (Figure 2).

In *T. auritus*, the microsatellites were also present on the W chromosome, although in a lesser amount compared to *L. reinhardti* (Figures 3, 4). The slight accumulation on the W chromosome exhibited only (C)_30_. The microsatellites (A)_30_, (GA)_15_, (GC)_15_ and (CAG)_10_ were present only in subtelomeric heterochromatin. Microsatellite (CAG)_10_ was strongly accumulated on one autosome pair. While the Z chromosome lacks 18S rDNA FISH signals, 18S rDNA sites were present on two autosomal pairs and on the W chromosome of *T. auritus*, where a strong accumulation of this sequence was found throughout its terminal heterochromatic region (Figure 3).

## Discussion

We studied the pattern of repeated DNAs distribution in the evolution of sex chromosomes in two fish species with ZW sex chromosome systems. In both species, the repetitive DNA has accumulated in the heterochromatic regions indicating that heterochromatinization has driven the divergence of Z and W chromosomes. We demonstrated a stronger microsatellite accumulation on the large W chromosome of *L. reinhardti* and only weak accumulation on the smaller W chromosome of *T. auritus*.

In *L. reinhardti,* a substantial accumulation of several microsatellite repeats on the W chromosome contributed to its enlargement in comparison to the Z chromosome (Figures 2,4). The accumulation of various types of repetitive DNA sequences on the W chromosome of other *Leporinus* species has also been documented [25,26]. Specifically in *L. elongatus*, a satellite DNA family named L6 was specific to the W chromosome [25], and a second one, Le*Spe* I, was a sex-specific dispersed repetitive element showing distinct distribution patterns on two exclusive female chromosomes, named W_1_ and W_2._ Therefore, it was suggested that instead of ZW sex chromosomes, *L. elongatus* may have a multiple Z_1_Z_1_Z_2_Z_2_ /Z_1_Z_2_W_1_W_2_ sex chromosome system [26].

Among *Triportheus* species, Z chromosome is conserved corresponding to the biggest one in the karyotype, while the W chromosome varies greatly between species concerning its size, morphology, and amounts of heterochromatin [24]. In *T. auritus*, in which the W chromosome is smaller than the Z chromosome, the accumulation of microsatellites was present only in one microsatellite class (Figures 3,4). The unique feature of *Triportheus* species W chromosomes is the presence of 18S rDNA at terminal region of the long arm [27]. Few examples of sex chromosomes bearing rRNA genes are known in fishes [28-31]. The redundancy of the rDNA sequences could make these chromosomal regions more susceptible to unequal crossing-over. In salmonid fishes, for example, an important role was suggested for the rDNA loci on the putative sex chromosomes of this species that might have limited the opportunity for additional recombination near a major sex-determining locus [28]. As the occurrence of 18S rDNA is a common feature for the W chromosomes in *Triportheus* species [27], a possible role for the repetitive DNAs associated with the rDNA, or even for the proper rDNA multicopy, in the differentiation of the W chromosome in this fish group could not be excluded.

In fact, variation in the amount of several types of repetitive DNA is associated with the genomic diversity and sex chromosome evolution of many fish species*.* For example, the Neotropical fish *Hoplias malabaricus* has different sex chromosome systems, as well as distinct evolutionary stages of sex chromosome differentiation found among its populations. In some populations of this species, a well-differentiated XX/XY sex chromosome system can be found in which the X chromosome clearly differs from the Y by the accumulation of DNA repeats [30,31]. At least 15 distinct repetitive DNA classes (including satellites, TEs, and microsatellites repeats) accumulated in the heterochromatic region of the X chromosome. Remarkably, in this case the X chromosome was the preferred site for the accumulation of the repeats, representing an unusual example of an X chromosome accumulating more repetitive DNA than the Y [31]. Studies conducted in phylogenetically distant organisms, such as the lizard *Eremias velox* (ZW system) and the plant *Silene latifolia* (XY system) showed extensive accumulation of several microsatellite sequences over the whole length or parts of the W and Y chromosomes, respectively. Accumulation of different microsatellites occurring on independently evolved sex chromosomes indicates that various repeated DNAs may follow very different “trajectories” on sex chromosomes in different lineages [32,33].

How is recombination suppressed? The accumulation of repetitive DNAs on the sex chromosomes may be both the cause, as well as the consequence of the recombination suppression. When the sex chromosomes stop their recombination, repetitive sequences are predicted to accumulate rapidly and this phenomenon may precede gene degeneration. Accumulated repetitive sequences may explain why the young Y chromosomes found in *Drosophila* and in some plants, are often larger than the X chromosomes [4,5]. However, the accumulation of repetitive DNAs on sex chromosomes cannot be taken as direct evidence for degeneration, as the accumulation does not automatically lead to gene loss. Studies conducted on some model species can reveal if the process of sex chromosomes evolution is, in fact, more dynamic than traditionally appreciated, helping us understanding how sex chromosomes become non-recombining, and the evolutionary transition from homologous to heteromorphic sex chromosomes.

The process of repetitive DNA accumulation probably represents the earliest events working on evolving sex-specific chromosomes before genes start to degenerate [34,35]. In this way, the W chromosome of *L. reinhardti* differ from that of *T. auritus* in the amount of accumulated DNA repeats, making it greater than the Z chromosome and, consequently, with a not degeneration in size. In this first species, the microsatellite repeats are the main class accumulated on the W chromosome, while in *T. auritus* such accumulation was no longer so prominent. Thus, in this latter species, other repeated DNA families may occupy this niche, representing the main component of the W chromosome heterochromatin, linked to its clear size reduction. In fact, large families of satellite DNAs can constitute the main component of the heterochromatin of the sex chromosomes, as found in birds and mammals [35-38], or even among fish, as appears to occur in *T. auritus.* Thus, microsatellite repeats seem to play a key role in the sex-specific chromosome differentiation, suggesting that they could be an early colonizer of sex chromosomes.

## Conclusions

The present data highlight that the evolution of the sex chromosomes can diverge even in the same type of sex system. In *L. reinhardti* and several other species of this same genus, as well as in the Parodontidae and Prochilodontidae families [39], the W chromosome notably increased in size compared to the Z chromosome. In contrast, in *T. auritus*, as well as in other species of this same genus or in the Crenuchidae family [39], the W chromosome behaves similarly as in higher vertebrates, showing size degeneration/shrinkage.

In both models, it is clear the close relationship between the differentiation of the W chromosome and its huge heterochromatinization. However, remains unanswered if there are cytological limitations involving the processes of growing or degeneration of the sex chromosomes. It’s not clear if the crescent accumulation of repetitive sequences in young sex chromosomes is necessarily followed by degeneration as commonly observed in higher vertebrates. In this sense, the accumulation or loss of repetitive sequences might have implications more than only quantitative. Could the destiny of the sex-specific chromosome follow alternative patterns, i.e. not necessarily being degenerated in size as commonly found in some groups? This question, however, only time and the evolution will tell us.

## Methods

### Specimens, mitotic chromosome preparation, chromosome staining, and karyotyping

Twelve females of *L. reinhardti* and fifteen females of *T. auritus* were collected from the São Francisco River (Minas Gerais State, Brazil) and Rio Negro River (Amazonas State, Brazil), respectively. Mitotic chromosomes were obtained from cell suspensions of the anterior kidney using the conventional air-drying method [40]. The experiments followed ethical protocols and anesthesia was administered prior to sacrificing the animals, according to the instructions of the local Ethical Committee. Chromosomes were sequentially Giemsa stained and C-banded using barium hydroxide [41] to detect the C-positive heterochromatin. Images were captured by an Olympus DP71 digital camera system (Olympus Corporation, Ishikawa, Japan) using the CoolSNAP system software, Image Pro Plus, 4.1 (Media Cybernetics, Silver Spring, MD, USA), coupled to an Olympus BX50 microscope (Olympus Corporation, Ishikawa, Japan). The chromosomes were classified as metacentric (m), submetacentric (sm), subtelocentric (st) or acrocentric (a) according to the arm ratios [42], and arranged in decreasing order of size in the karyotype.

### Fluorescence in situ hybridization of repetitive DNAs on mitotic spreads

Nine repetitive DNA sequences were used as probes, including an 18S rDNA gene and eight microsatellite repeats. The 18S rDNA probe corresponded to a 1400 bp-segment of the 18S rRNA gene, obtained via PCR from nuclear DNA, previously cloned into plasmid vectors and propagated in *Escherichia coli* DH5α [43]. This probe was labeled with digoxigenin-11-dUTP by nick translation, following the manufacturer’s instructions (Bionick Labeling System, Invitrogen). Fluorescence in situ hybridization (FISH) was performed according to [44] and the detection of the 18S rDNA hybridization signals were performed using anti-digoxigenin-fluorescein (Roche, Mannheim, Germany). FISH experiments with the microsatellite probes were performed as described in [32], with slight modifications. We used the following labeled oligonucleotides as probes: d(A)_30_, d(C)_30_, d(CA)_15_, d(GA)_15_, d(GC)_15_, d(TA)_15_, d(CAT)_10_, d(CAG)_10_. These sequences were directly labeled with Cy3 at 5´ terminal during synthesis by Sigma (St. Louis, MO, USA). The chromosomes were counterstained with DAPI (1.2 μg/ml), mounted in antifade solution (Vector, Burlingame, CA, USA), and analyzed in an epifluorescence microscope Olympus BX50 (Olympus Corporation, Ishikawa, Japan).

## Abbreviations

2n: Diploid number; A: Acrocentric chromosome; DAPI 4': 6-diamidino-2-phenylindole; dATP 2': Deoxyadenosine triphosphate; FISH: Fluorescence in situ hybridization; M: Metacentric chromosome; PCR: Polymerase chain reaction; rDNA: Ribosomal DNA; rRNA: Ribosomal RNA; sm: Submetacentric chromosome; st: Subtelocentric chromosome; TEs: Transposable elements.

## Competing interests

The authors declare that they have no competing interests

## Authors’ contributions

MBC coordinated the study, carried out the molecular cytogenetic analysis, and drafted the manuscript. JP and VM carried out the conventional and molecular cytogenetic analysis and drafted the manuscript. EK, DD and WFM helped in analysis and drafted the manuscript. LACB drafted and revised the manuscript. All authors read and approved the final version of the manuscript.
