# Correction: The key role of repeated DNAs in sex chromosome evolution in two fish species with ZW sex chromosome system

**DOI:** 10.1186/1755-8166-5-42

**Published:** 2012-11-27

**Authors:** Marcelo de Bello Cioffi, Eduard Kejnovský, Vinicius Marquioni, Juliana Poltronieri, Wagner F Molina, Débora Diniz, Luiz Antonio C Bertollo

**Affiliations:** 1 Departamento de Genética e Evolução, Universidade Federal de São Carlos, São Carlos, SP, Brazil; 2 Department of Plant Developmental Genetics, Institute of Biophysics ASCR, Brno, Czech Republic; 3 Laboratory of Genome Dynamics, CEITEC - Central European Institute of Technology, Masaryk University, Brno, Czech Republic; 4 Departamento de Biologia Celular e Genética, Centro de Biociências, Universidade Federal do Rio Grande do Norte, Natal, RN, Brazil; 5 Departamento de Ciências Biológicas, Universidade Estadual do Sudoeste da Bahia, Jequié, BA, Brazil

## Correction

After the publication of this work [1] the following errors were brought to the authors’ attention: Figure 1 contained a misspelling of the species name *Leporinus reinhardti,* and Figures 2 and 3 contained mistakes that occurred during the editing process. The correct figures are given below. 

**Figure 1:**
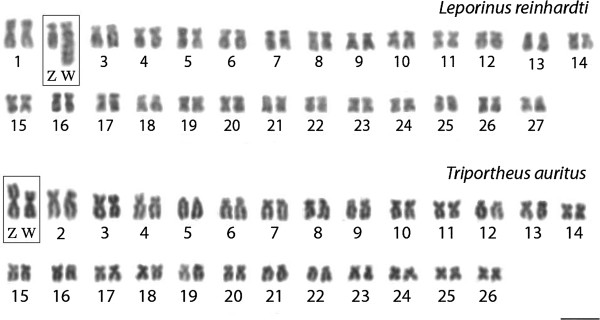
**Giemsa-stained female karyotypes of *Leporinus reinhardti *(2n = 54) and *Triportheus auritus *(2n = 52), both with a ZZ/ZW sex chromosome system.** The chromosomes of both species were arranged in descending order of size and the sex chromosomes were highlighted in boxes for the sake of clarity. Bar = 5 μm.

**Figure 2:**
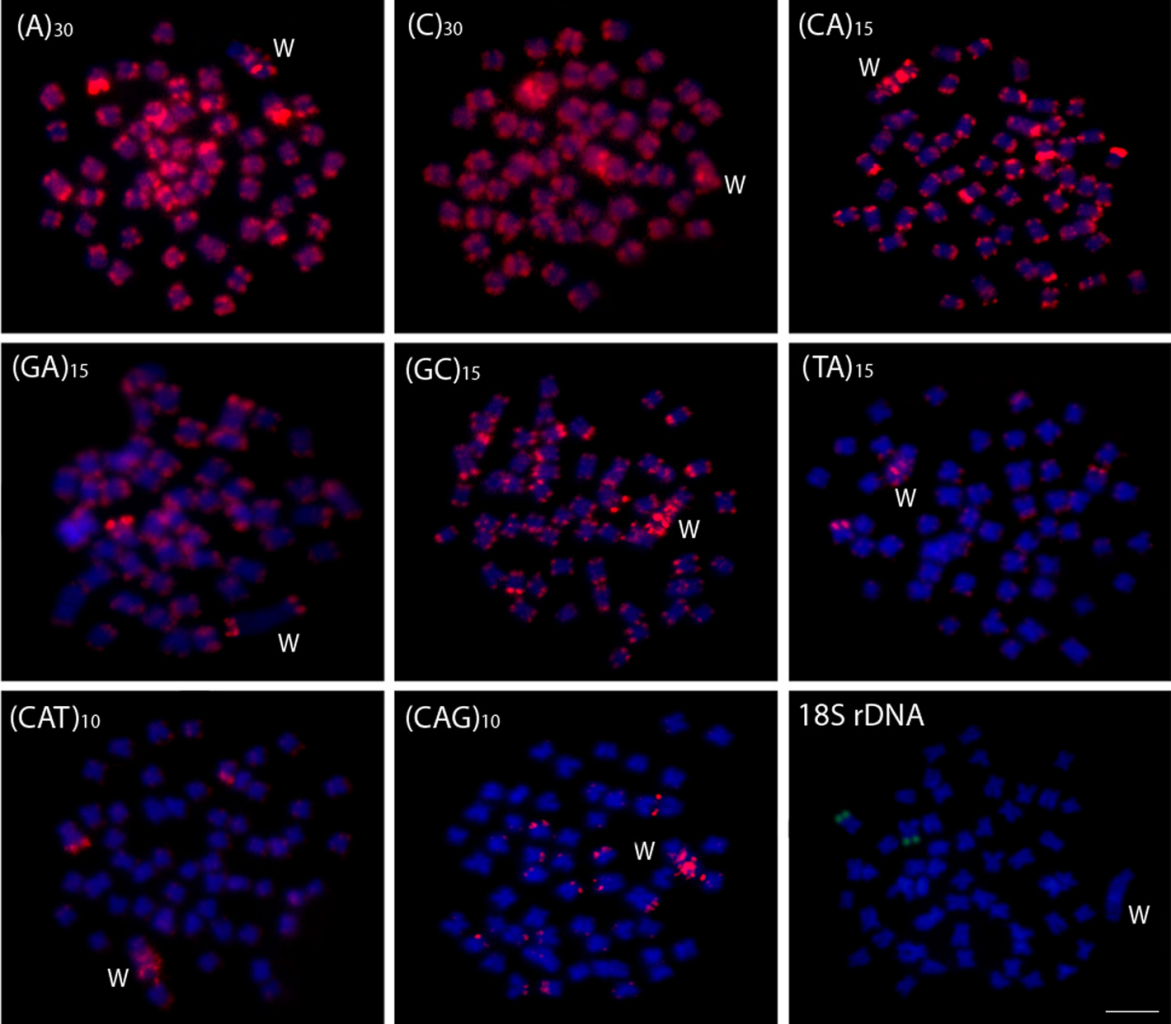
**Mitotic metaphase chromosomes of *Leporinus reinhardti *female, with a ZZ/ZW sex chromosome system hybridized with different repeated DNAs, including mono-, di- and trinucleotide microsatellites and an 18S rDNA gene as probes.** Letters mark the W chromosomes. Bar = 5 μm.

**Figure 3:**
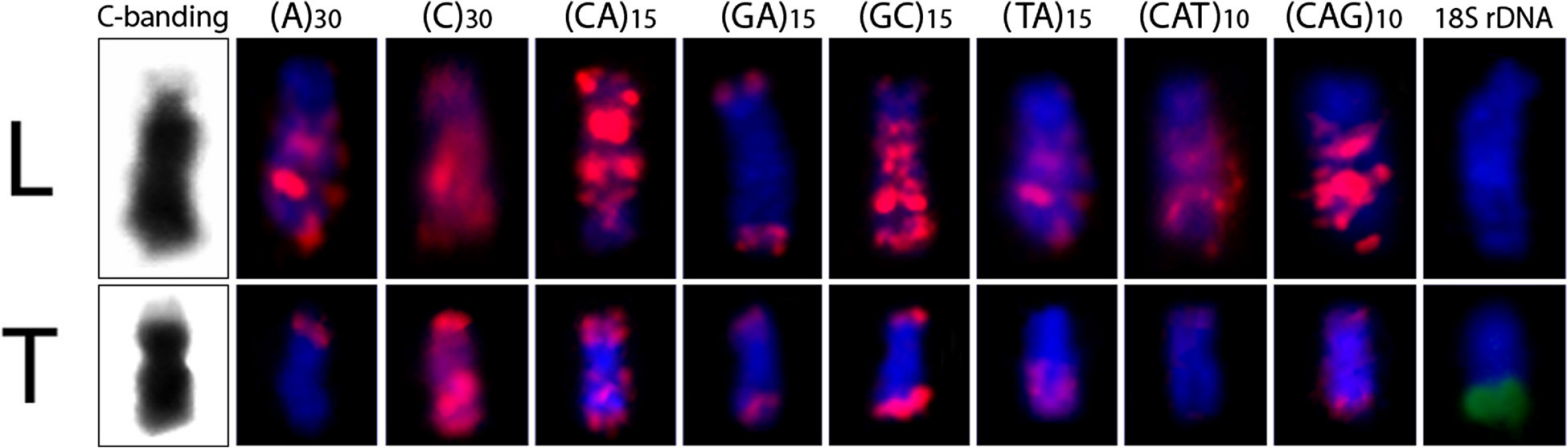
**W chromosomes of *Leporinus reinhardti *(L) and *Triporteus auritus *(T) after C-banding and FISH with various repetitive DNA sequences.** Note the huge accumulation of several classes of microsatellites in *L. reinhardti* and the lesser amount of this accumulation in *T. auritus*.

We regret any inconvenience that this inaccuracy may have caused.
